# Random measurement and prediction errors limit the practical relevance of two velocity sensors to estimate the 1RM back squat

**DOI:** 10.3389/fphys.2024.1435103

**Published:** 2024-09-10

**Authors:** Konstantin Warneke, Josua Skratek, Carl-Maximilian Wagner, Klaus Wirth, Michael Keiner

**Affiliations:** ^1^ Institute of Human Movement Science, Sport and Health, University of Graz, Graz, Austria; ^2^ Department for Sport and Exercise Science, German University of Health and Sport, Ismaning, Germany; ^3^ Institute of Movement, Sport and Health, Leuphana University Lüneburg, Lüneburg, Germany; ^4^ Department for Sport Science, University of Applied Sciences Wiener Neustadt, Wiener Neustadt, Austria

**Keywords:** velocity profile, velocity-based training, strength estimation, measurement error, reliability

## Abstract

**Introduction:**

While maximum strength diagnostics are applied in several sports and rehabilitative settings, dynamic strength capacity has been determined via the one-repetition maximum (1RM) testing for decades. Because the literature concerned several limitations, such as injury risk and limited practical applicability in large populations (e.g., athletic training groups), the strength prediction via the velocity profile has received increasing attention recently. Referring to relative reliability coefficients and inappropriate interpretation of agreement statistics, several previous recommendations neglected systematic and random measurement bias.

**Methods:**

This article explored the random measurement error arising from repeated testing (repeatability) and the agreement between two common sensors (vMaxPro and TENDO) within one repetition, using minimal velocity thresholds as well as the velocity = 0 m/s method. Furthermore, agreement analyses were applied to the estimated and measured 1RM in 25 young elite male soccer athletes.

**Results:**

The results reported repeatability values with an intraclass correlation coefficient (ICC) = 0.66–0.80, which was accompanied by mean absolute (percentage) errors (MAE and MAPE) of up to 0.04–0.22 m/s and ≤7.5%. Agreement between the two sensors within one repetition showed a systematic lower velocity for the vMaxPro device than the Tendo, with ICCs ranging from 0.28 to 0.88, which were accompanied by an MAE/MAPE of ≤0.13 m/s (11%). Almost all estimations systematically over/ underestimated the measured 1RM, with a random scattering between 4.12% and 71.6%, depending on the velocity threshold used.

**Discussion:**

In agreement with most actual reviews, the presented results call for caution when using velocity profiles to estimate strength. Further approaches must be explored to minimize especially the random scattering.

## 1 Introduction

Competitive team sports, such as soccer, require a combination of technical, tactical, and physical abilities (Stølen et al., 2005). Although sprinting only accounts for up to 3% of effective playtime and approximately 10% of total distance in a typical soccer match, players perform approximately 1,300 speed and strength actions, such as jumping and linear and change of direction sprints (Bangsbo et al., 2006; Stølen et al., 2005). To achieve superior speed strength performances, it is necessary to generate the largest possible ground reaction forces by the neuromuscular system within short ground contact times. Therefore, literature reported high correlates between maximum strength and speed performance (Keiner et al., 2022; Suchomel et al., 2016; Warneke et al., 2022), suggesting resistance training could effectively improve sprinting and jumping ability (Keiner et al., 2014; Lohmann et al., 2022; Wirth et al., 2016).

Using a percentage of the one-repetition maximum (1RM) to regulate training intensity is a popular method for measuring maximum strength. However, applying this technique may present difficulties for both coaches and athletes, especially those with less experience. Percentage-based training (PBT) requires accurate testing of the 100% 1RM before using high-load, 1RM normalized training protocols (i.e., loads of 80% of the 1RM) (Włodarczyk et al., 2021). Especially in large group constellations such as athletic training or testing, literature concerns limited practicability due to insufficient supervision to fix incorrect movement execution that might cause an increased injury risk (Eston and Evans, 2009; González-Badillo and Sánchez-Medina, 2010; Jovanovic and Flanagan, 2014). Furthermore, critics of 1RM tests argue that maximum strength values possess a high variability, depending on the athlete’s daily form or a lack of familiarity with 1RM testing in the intended exercises (Benton et al., 2009).

To provide valuable and reliable alternatives to conduct maximal strength testing periodically and to save time and avoid fatigue (González-Badillo and Sánchez-Medina, 2010; Jovanovic and Flanagan, 2014), a growing body of evidence supports the utilization of velocity sensors such as the vMaxPro (Dragutinovic et al., 2024; Feuerbacher et al., 2022; Thompson et al., 2020) or the Tendo (Suchomel et al., 2023) that track the velocity of the bar in submaximal trials to estimate the participants’ 1RM by using the velocity bar profile for linear regression (González-Badillo and Sánchez-Medina, 2010; Pestaña-Melero et al., 2018). While assumed reliable (Clemente et al., 2021; Orange et al., 2019; Orange et al., 2020) and effective in training (Zhang et al., 2022), the stated reliability with an intraclass correlation coefficient (ICC) = 0.65–0.99 provides a broad range of reliability effect sizes. Especially for the vMaxPro, Dragutinovic et al. (2024) and Feuerbacher et al. (2022) reported practical limitations due to reduced reliability in high velocities (light weights), while inter-day reliability was reported with an ICC = 0.57–0.84, which is classified as moderate to good (Shrout and Fleiss, 1979).

While literature generally supports VBT application despite acknowledging validity concerns (Guppy et al., 2023), depending on the chosen model, it often focuses on relative reliability metrics like ICC and Pearson coefficients, overlooking their limitations in detecting measurement errors, as noted by Atkinson and Nevill (1998) and Hopkins (2000). While Bland–Altman (BA) plots are frequently used in device validation studies, Grgic et al. (2020) requested the implementation in reliability research as well. Lin (1989) recommended both the concordance correlation coefficient (CCC) and BA analysis for precise bias assessment (Bland and Altmann, 1986; Doğan, 2018). The infrequent application of these tools in existing literature could lead to misjudgments. Additionally, the integration of mean absolute error (MAE) (C. Willmott and Matsuura, 2005) and mean absolute percentage error (MAPE) provides a detailed examination of the unsystematic bias, which may elucidate the observed variability in VBT reliability evaluations.

Consequently, to counteract the listed limitations, this work will first focus on quantifying the measurement error arising from repeated measures to provide an advanced expectation of individual error scattering. Second, assuming the ability to appropriately track the velocity of the bar, an agreement analysis is performed between two sensors attached to the same bar. Third, the agreement between the estimated 1RM of the vMaxPro and the Tendo device with the tested back squat 1RM is determined. For a valuable practical use, it must be hypothesized that both sensors would agree on the tracked movement velocity (valid to produce load-velocity profiles) and will be able to estimate the back squat 1RM without a systematic and random bias.

## 2 Methods

### 2.1 Experimental approach to the problem

The objective of the study was to perform an agreement analysis between dynamic free-weight maximal strength measurements (1RM) and velocity-based maximal strength estimations. To answer the research question, 25 elite youth soccer players competing in the respective highest national league were recruited. The test protocol was divided into 2 testing days. Test day 1 consisted of 1RM in the squat. One week after the 1RM testing, two frequently used velocity sensors were attached to a straight bar. The participants were instructed to perform two trials of the parallel back squat as fast as possible with a prescribed percentage of their respective parallel 1RM back squat. All participants were familiar with maximal strength testing of the 1RM back squat and usually performed parallel squat training in the regular strength and conditioning training regime. In order to familiarize the participants with load–velocity profiling, each athlete completed the load–velocity profiling protocol without using attached VBT devices following the 1RM tests. No training sessions were conducted on the day prior to the respective test days, and participants were instructed to avoid strenuous activities. On test days, participants refrained from training before testing. Throughout all tests, researchers provided strong verbal encouragement to ensure participants exerted maximal effort. To minimize potential confounding factors, participants were instructed to maintain their regular dietary habits and fluid intakes and to refrain from consuming caffeine and other neurostimulants in the 3 h leading up to each laboratory test. All tests were conducted at approximately the same time of the day to avoid circadian variance within a range of ±1.5 h.

### 2.2 Participants

The study included twenty-five (25) national-level (Tier 3) youth male soccer players from two teams (U17 [under 17 years], U19) of a youth elite training center associated with a professional club in the third division in Germany. The U17 and U19 youth soccer teams played in the highest German leagues (Bundesliga), respectively. The mean ± standard deviation [SD] (confidence interval [CI]) characteristics of the group were as follows: age: 16.8 ± 1.35 years; height: 180.45 ± 5.30 cm; body mass: 70.68 ± 6.62 kg. Their training during the period of testing consisted of five training sessions per week with competitions on weekends (8 h training/week). The training sessions consisted of team and position-specific soccer training, as well as strength and conditioning training, including resistance training and plyometric exercises (i.e., jumping and sprinting). All subjects were regular starters and competed with their teams in their respective leagues on weekends during the season. The soccer players were classified as elite in reference to the definition used by Lorenz et al. (2013), who considered elite athletes as those who played at a higher level than peers within a sport. All participants and the parents of those under the age of 18 provided written informed consent to participate. The study was approved by the local university’s institutional ethics committee (DHGS-EK- 2021-002), and study procedures adhered to the principles outlined in the Declaration of Helsinki.

### 2.3 One-repetition maximum test

Maximal strength was assessed using the one-repetition maximum (1RM) in the high bar back squat, with a 20-kg barbell positioned on the ascending trapezius muscle. Participants were free to choose a self-selected foot position and eccentric speed. Repetition maximum testing was conducted in accordance with the guidelines established by the National Strength and Conditioning Association (Haff and Triplett, 2016). The warm-up protocol included a 10-min warm-up on a cycle ergometer, followed by three sets with 2–5 repetitions at approximately 50%–80% of 1RM for each exercise. The initial attempts were performed with a load of approximately 90%–95% of the estimated 1RM (based on commonly used training loads). After each successful attempt, the load was increased by 2%–5% until participants failed to lift the load with a proper technique. Rest periods of at least 5 min were provided between trials, and 1RMs were achieved within a maximum of five attempts. Squat depth was visually assessed and verbally reinforced by the same trained investigator (i.e., a master sports science certified strength and conditioning coach with 5 years of experience). The required squatting depth was reached when the hip joint was deeper than the femur. A brief hold (approx. 1 s) after the eccentric phase was implemented to avoid the utilization of the rebound effect at the bottom of the squat movement. Squat attempts failed if participants could not stabilize the bar with their backs, lost the bar, or could not achieve the required depth. The highest accepted attempt after two consecutive non-accepted attempts was registered as the 1RM. A high ICC of 0.91–0.99, as a measure of test-retest reliability, has been reported in previous research (Keiner et al., 2021; McMaster et al., 2014).

### 2.4 Load–velocity profile

The mean velocity of all repetitions was monitored using two mobile systems: a wireless inertial measurement unit (VMaxPro sensor, Blaumann and Meyer Sports Technology UG, Magdeburg, Germany) with a sampling rate of 200 Hz and a rotatory encoder with cable extension (Tendo Uni, TENDO Sport, Trencin, Slovak Republic). The rotary encoder precisely recorded the position and direction of the bar with an accuracy of 0.3 mm. According to the manufacturer’s instructions, the devices were placed between the hands and the barbell sleeves on both sides of the barbell, respectively. Data obtained from both devices were transmitted via Bluetooth and evaluated via custom software, the Enode app Version 2.1.1. and the Tendon app Version PA7.1.4. The mean velocity of all trials was manually recorded in a Microsoft Excel spreadsheet (Microsoft Corporation, Redmond, Washington, United States). Following the standardized warm-up used in the 1RM session, load–velocity profiling was conducted using loads corresponding to 20%, 40%, 60%, 80%, and 90% of the previously determined 1RM. Two concentric actions were recorded for each load, and the one with the highest absolute mean velocity value was selected for further analysis. The rest interval between each trial was 2 min. Participants were instructed to exert maximum force and speed during the concentric phase of the squatting movement while adhering to the same movement pattern performed during 1RM testing. To avoid differences in bar displacement, the investigator who supervised the 1RM tests also visually assessed and verbally reinforced squatting depth. A brief rest period of approximately 1 s was implemented after the eccentric muscle action and before the concentric muscle action to prevent any eccentric-concentric movement coupling.

### 2.5 Data processing and statistical analysis

The data analysis was performed using JAMOVI (Version 2.4.7), using the regression-, seolmatrix-, blandr-, and SimpleAgree packages. Normal data distribution was ensured using the Shapiro–Wilk test. The descriptive statistics of the 1RM weight from each trial, submaximal loads (20%, 40%, 60%, 80%, and 90% 1RM), as well as the respective average velocity were provided via M ± standard deviation (SD). Intraday reliability was evaluated between the two separated trials, and relative reliability analysis using ICCs for agreement was supplemented by the concordance correlation coefficient (Lin, 1989) as well as an agreement analysis with Bland–Altman plots (Doğan, 2018), with quantified lower and upper limits of agreement as well as the systematic bias (mean differences between the first and the second trial. The systematic bias was assessed and tested for significance using the dependent t-test. To avoid type 1 error accumulation, alpha error correction via Bonferroni was applied. The qualitative random scattering inspection was completed by calculating the MAE (Willmott and Matsuura, 2006; Willmott and Matsuura, 2005), with $MAE=\frac{1}{n}*\sum_{i=1}^{n}\left|x_{i}-y_{i}\right|$ , and the MAPE (Hyndman and Koehler, 2006; Kim and Kim, 2016; Makridakis, 1993), with $\text{MAPE}=\frac{1}{n}*\sum_{i=1}^{n}\left|\frac{x_{i}-y_{i}}{x_{i}}\right|*100$ , using the first trial as the reference. Both parameters are reported to investigate the difference between a measured and predicted parameter when validating testing batteries. Reliability analysis was performed for the VMaxPro and the Tendo mean velocities.

Because both sensors were attached to the same bar, it was hypothesized that both would measure the same velocity. Deviations were also calculated using the CCC, BA analysis, MAE, and MAPE.

Assuming a linear relationship between velocity and intensity (Loturco et al., 2021; Ruf et al., 2018), a bivariate two-tailed test was applied. In the light of practical applicability, a high agreement between the measured 1RM and the estimated 1RM must be assumed as well. Thus, the agreement analysis was performed for the vMaxPro and Tendo calculations, respectively, on previously determined 1RM to investigate the validity of the velocity profile based on estimated 1RM from the intern vMaxPro software, the Vmin model using v = 0.3 (Guppy et al., 2023), and the actually measured 1RM.

## 3 Results

Normal distribution of data was ensured with *p* > 0.05. Mean velocities for 20% ranged from 1.14 ± 0.08 m/s to 1.28 ± 0.11 m/s, dependent on trial and measurement devices. With increasing load to 90% 1RM, the velocity dropped to a minimum of 0.60 ± 0.07 m/s (Tables 1, 2).

**TABLE 1:** Repeatability of the testing, providing the relative reliability and the systematic and random bias resulting from repeated measurements.

Parameter	Value 1 in m/s	Value 2 in m/s	ICC (95%CI)	Concordance r	MAE in m/s	MAPE in %	Mean diff. (*p*-value)
20% MVvMaxPro T1 vs. MVvMaxPro T2	1.14 ± 0.08	1.17 ± 0.09	0.68; (0.51–0.89)	0.67; (0.41–0.83)	0.06	5.3	0.04 (0.008)
20% MVTendo T1 vs. MVTendo T2	1.23 ± 0.13	1.28 ± 0.11	0.66; (0.50–0.86)	0.65; (0.39–0.82)	0.07	6.5	0.05 (0.006)
40% MVvMaxPro T1 vs. MVvMaxPro T2	0.98 ± 0.07	1.01 ± 0.08	0.78; (0.61–1.00)	0.77; (0.58–0.89)	0.04	3.8	0.02 (0.025)
40% MVTendo T1 vs. MVTendo T2	1.06 ± 0.10	1.08 ± 0.09	0.74; (0.48–1.00)	0.73; (0.49–0.87)	0.04	4.2	0.02 (0.233)
60% MVvMaxPro T1 vs. MVvMaxPro T2	0.83 ± 0.07	0.85 ± 0.06	0.79; (0.61–1.00)	0.78; (0.58–0.90)	0.03	4.1	0.01 (0.18)
60% MVTendo T1 vs. MVTendo T2	0.90 ± 0.08	0.89 ± 0.06	0.70; (0.42–1.00)	0.69; (0.44–0.85)	0.04	4.4	0.01 (0.35)
80% MVvMaxPro T1 vs. MVvMaxPro T2	0.67 ± 0.05	0.68 ± 0.04	0.80; (0.70–0.93)	0.80; (0.61–0.90)	0.22	3.5	0.009 (0.13)
80% MVTendo T1 vs. MVTendo T2	0.70 ± 0.06	0.70 ± 0.06	0.77; (0.57–1.00)	0.76; (0.53–0.89)	0.03	4.7	0.002 (0.86)
90% MVvMaxPro T1 vs. MVvMaxPro T2	0.61 ± 0.07	0.60 ± 0.07	0.81; (0.56–1.00)	0.80; (0.61–0.91)	0.03	5.3	0.01 (0.22)
90% MVTendo T1 vs. MVTendo T2	0.64 ± 0.07	0.62 ± 0.08	0.74; (0.55–1.00)	0.73; (0.49–0.87)	0.05	7.5	0.02 (0.15)

MV = mean velocity, VMaxPro = VMaxPro velocity testing device from Enode, Tendo = cable-based velocity sensor, T1 = trial 1, T2 = trial 2, ICC = intraclass correlation coefficient, MAE = mean absolute error, MAPE = mean absolute percentage error, concordance r = concordance correlation coefficient, 20% = 20% of the previously determined 1RM, 40% = 40% of the previously determined 1RM, 60% = 60% of the previously determined 1RM, 80% = 80% of the previously determined 1RM, 90% = 90% of the previously determined 1RM, * = significant after Bonferroni correction (threshold = 0.005).

**TABLE 2:** Agreement in the velocity determination between the vMaxPro and the Tendo device for the first and second trials and the best results extracted out of both trials.

Parameter	Velocity vMaxPro in m/s	Velocity Tendo in m/s	Correlation coefficient (*p*-value)	ICC (95%CI)	Concordance r (95%CI)	MAE in m/s	MAPE in %	Mean diff. (*p*-value)
20% vMaxPro1 vs. Tendo1	1.2 ± 0.09	1.2 ± 0.13	0.55 (0.004)	0.37; (0.26–0.50)	0.36; (0.11–0.57)	0.13	11.0	0.09 (<0.001)*
20% vMaxPro2 vs. Tendo2	1.17 ± 0.09	1.3 ± 0.13	0.86 (<0.001)	0.53; (0.40–0.70)	0.52; (0.32–0.68)	0.11	9.8	0.11 (<0.001)*
20% vMaxPro best vs. Tendo best	1.18 ± 0.09	1.29 ± 0.10	0.82 (<0.001)	0.52; (0.37–0.70)	0.51; (0.30– 0.67)	0.11	9.4	0.10 (<0.001)*
40% vMaxPro1 vs. Tendo1	1.01 ± 0.08	1.09 ± 0.09	0.88 (<0.001)	0.59; (0.46–0.75)	0.58; (0.38–0.72)	0.09	8.7	0.08 (<0.001*
40% vMaxPro2 vs. Tendo2	1.01 ± 0.08	1.08 ± 0.09	0.83 (<0.001)	0.62; (0.48–0.80)	0.61; (0.39–0.76)	0.08	7.8	0.07 (<0.001)*
40% vMaxPro best vs. Tendo best	1.01 ± 0.08	1.09 ± 0.09	0.82 (<0.001)	0.60; (0.45–0.78)	0.59; (0.37–0.75)	0.08	8.3	0.07 (<0.001)*
60% vMaxPro1 vs. Tendo1	0.85 ± 0.06	0.91 ± 0.06	0.80 (<0.001)	0.53; (0.32–0.80)	0.52; (0.30–0.69)	0.07	9.0	0.07 (<0.001)*
60% vMaxPro2 vs. Tendo2	0.84 ± 0.06	0.89 ± 0.06	0.86 (<0.001)	0.65; (0.49–0.86)	0.64; (0.43–0.78)	0.05	6.2	0.05 (<0.001)*
60% vMaxPro best vs. Tendo best	0.85 ± 0.06	0.91 ± 0.06	0.70 (<0.001)	0.50; (0.25–0.83)	0.49; (0.24–0.68)	0.06	7.3	0.06 (<0.001)*
80% vMaxPro1 vs. Tendo1	0.69 ± 0.04	0.72 ± 0.06	0.32 (0.114)	0.28; (−0.06–0.60)	0.28; (−0.67–0.58)	0.05	8.5	0.03 (0.033)
80% vMaxPro2 vs. Tendo2	0.68 ± 0.04	0.70 ± 0.06	0.56 (0.004)	0.49; (0.27–0.73)	0.48; (0.18–0.69)	0.05	6.7	0.02 (0.079)
80% vMaxPro best vs. Tendo best	0.69 ± 0.04	0.72 ± 0.06	0.49 (0.013)	0.40; (0.15–0.68)	0.39; (0.09–0.63)	0.05	7.3	0.03 (0.026)
90% vMaxPro1 vs. Tendo1	0.61 ± 0.07	0.64 ± 0.08	0.87 (<0.001)	0.80; (0.65–1.00)	0.80; (0.62–0.90)	0.04	6.7	0.03 (<0.001)*
90% vMaxPro2 vs. Tendo2	0.60 ± 0.07	0.62 ± 0.08	0.85 (<0.001)	0.80; (0.59–1.00)	0.79; (0.61–0.89)	0.03	5.6	0.02 (0.013)
90% vMaxPro best vs. Tendo best	0.61 ± 0.07	0.64 ± 0.08	0.85 (<0.001)	0.76; (0.58–1.00)	0.75; (0.55–0.87)	0.04	7.5	0.03 (<0.001)*

VMaxPro = VMaxPro velocity testing device from Enode, Tendo = cable-based velocity sensor, T1 = trial 1, T2 = trial 2, ICC = intraclass correlation coefficient, MAE = mean absolute error, MAPE = mean absolute percentage error, concordance r = concordance correlation coefficient, 20% = 20% of the previously determined 1RM, 40% = 40% of the previously determined 1RM, 60% = 60% of the previously determined 1RM, 80% = 80% of the previously determined 1RM, 90% = 90% of the previously determined 1RM, * = significant after Bonferroni correction (threshold = 0.003).

### 3.1 Reliability

Relative reliability was stated via ICC and ranged from 0.66 (20% 1RM measured via Tendo) to 0.81 (90% 1RM measured via vMaxPro), with CCCs of 0.65–0.80. With mean value references of 1.28 ± 0.11 m/s to 0.60 ± 0.07 m/s, the MAEs ranging from 0.03–0.22 correspond to MAPEs of 3.8%–7.5% arising from repeating the test. There was a significant systematical bias (*p* = 0.006–0.03) only for low loads and high velocities (20% 1RM, 40% 1RM), indicating the first trial was slower than the second trial with a mean difference of 0.02–0.05 m/s (Table 1).

### 3.2 Agreement between the two velocity devices in determining the bar velocity

Apart from the first trial with 80% 1RM, correlation coefficients showed a moderate to high association (r = 0.50–0.88) between both devices. Using agreement ICCs and the CCC showed relative reliabilities ranging between 0.36 and 0.80. Evaluating the systematic bias showed a significant difference for all but the vMaxPro vs. Tendo in the second trial with 80% 1RM, indicating systematically higher measured velocities in the Tendo. The random scattering around the systematic measurement error is reported with an MAE between 0.03 and 0.13, which can be expressed with an MAPE of up to 11% (Table 2), with a maximum of 44% error between vMaxPro and Tendo in the first trial for 80% 1RM. Because this error seems to be an outlier, the second-highest MPE still provided a discrepancy between both velocity measurement devices of 27.4% (second trial with 90% 1RM).

### 3.3 Validity of load–velocity profiles from the two velocities to estimate the 1RM

Correlation coefficients between the estimated models of the vMaxPro Software and the linear regression models for v = 0.3 (Guppy et al., 2023) and v = 0.5 showed constant high correlation coefficients (rp = 0.79–0.82, *p* < 0.001). The ICCs for agreement, as well as the Lins CCC (Lin, 1989), ranged from 0.23 to 0.83, whereas the lowest values were reported for the v = 0.3 model (0.23–0.35), while the v = 0.5 ranged between 0.60 and 0.82. Accordingly, the MAEs and MAPEs for the v = 0.3 m/s model ranged from 28.10 kg to 36.22 kg and from 26.10% to 36.45%, respectively, and were reduced for the v = 0.5 m/s model (9.3–14.7 kg, 8.4%–14.6%). All but the estimated values of the vMaxPro under consideration of the RPE and the v = 0.5 m/s (Tendo and vMaxPro) showed a systematic bias, indicating significantly lower estimated values from the vMaxPro internal software than the v = 0.3 and v = 0.5 m/s regression models, independent of the sensor used (Table 3).

**TABLE 3:** Agreement between (a) estimated 1RM with and without RPE (vMaxPro) and linear regression model using Vo and (b) Vmin with the Tendo device, (c) measured 1RM and estimated 1RM with and without RPE (vMaxPro), (d) measured 1RM and linear regression model using V = 0.5, and (e) V = 0.3 with the 1) vMaxPro and 2) Tendo device.

Parameter		Value 1	Value 2	Correlation coefficient	ICC; (95%CI)	Concordance r; (95%CI)	MAE	MAPE	MPE	Systematic bias
Value 1	Value 2									
vMaxPro	Tendo V0	99.4 ± 14.26	170.55 ± 30.67	0.79 (<0.001)	0.11; (0.06–0.17)	0.11; (0.04–0.17)	71.15	71.63	108.7	−71.2 (<0.001)*
	Tendo V0.3		135.35 ± 23.9	0.80 (<0.001)	0.27; (0.16–0.39)	0.26; (0.12–0.39)	36.22	36.45	64.3	36.0 (<0.001)*
	Tendo V0.5		111.89 ± 19.73	0.81 (<0.001)	0.61; (0.43–0.82)	0.60; (0.38–0.76)	14.66	14.63	36.9	12.5 (<0.001)*
	vMaxPro V0		176.31 ± 27.55	0.77 (<0.001)	0.09; (0.05–0.14)	0.09; (0.32–0.14)	76.91	77.89	112.4	−76.9 (<0.001)*
	vMaxPro V0.3		136.58 ± 20.7	0.80 (<0.001)	0.24; (0.14–0.35)	0.23; (0.10–0.35)	37.18	37.80	56.97	37.2 (<0.001)*
	vMaxPro V0.5		110.10 ± 16.5	0.82 (<0.001)	0.66; (0.49–0.87)	0.65; (0.42–0.80)	12.75	13.00	26.72	10.7 (<0.001)*
vMaxProRPE	Tendo V0	108.24 ± 17.14	170.55 ± 30.67	0.77 (<0.001)	0.16; (0.09–0.23)	0.15; (0.06–0.24)	62.31	57.89	91.70	62.3 (<0.001)*
	Tendo V0.3		135.35 ± 23.92	0.79 (<0.001)	0.41; (0.28–0.57)	0.40; (0.21–0.56)	28.10	26.10	45.5	27.1 (<0.001)*
	Tendo V0.5		111.89 ± 19.73	0.82 (<0.001)	0.80; (0.66–0.99)	0.80; (0.61–0.90)	9.29	8.37	27.8	3.65 (0.12)
	vMaxPro V0		176.31 ± 27.55	0.73 (<0.001)	0.12; (0.06–0.19)	0.12; (0.04–0.19)	68.07	63.76	106.04	−68.1 (<0.001)*
	vMaxPro V0.3		136.58 ± 20.7	0.79 (<0.001)	0.37; (0.23–0.53)	0.35; (0.18–0.51)	28.98	27.35	52.26	28.3 (<0.001)*
	vMaxPro V0.5		110.10 ± 16.5	0.82 (<0.001)	0.83; (0.65–0.92)	0.82; (0.63–0.92)	7.05	6.46	26.30	1.86 (0.36)
1RM	vMaxPro	106.0 ± 15.68	99.4 ± 14.26	0.90 (<0.001)	0.82; (0.70–0.98)	0.81; (0.65–0.90)	7.72	7.15	18.3	6.60 (<0.001)*
	vMaxProRPE		108.24 ± 17.14	0.94 (<0.001)	0.93; (0.87–0.99)	0.92; (0.84–0.97)	4.32	4.12	17.0	2.24 (0.08)
	Tendo V0		170.55 ± 30.67	0.87 (<0.001)	0.16; (0.11–0.22)	0.15; (0.07–0.23)	64.55	60.74	93.22	64.6 (<0.001)*
	Tendo V0.3		135.35 ± 23.92	0.90 (<0.001)	0.40; (0.31–0.52)	0.40; (0.23–0.54)	29.35	27.53	46.6	29.4 (<0.001)*
	Tendo V0.5		111.89 ± 19.73	0.92 (<0.001)	0.85; (0.79–0.93)	0.84; (0.71–0.92)	8.72	8.30	15.7	5.89 (0.002)*
	vMaxPro V0		176.31 ± 27.55	0.85 (<0.001)	0.12; (0.08–0.18)	0.12; (0.05–0.19)	70.31	66.71	96.23	70.3 (<0.001)*
	vMaxPro V0.3		136.58 ± 20.7	0.89 (<0.001)	0.36; (0.63–0.48)	0.35; (0.19–0.49)	30.58	29.12	45.01	30.6 (<0.001)*
	vMaxPro V0.5		110.10 ± 16.5	0.92 (<0.001)	0.89; (0.83–0.96)	0.88; (0.76–0.95)	6.59	6.50	22.45	4.10 (0.005)
vMaxPro	vMaxProRPE	99.4 ± 14.26	108.24 ± 17.14	0.92 (<0.001)	0.78; (0.68–0.92)	0.78; (0.62–0.88)	9.0	9.10	22.4	8.84 (<0.001)*

VMaxPro = VMaxPro velocity testing device from Enode, Tendo = cable-based velocity sensor, V0 = velocity threshold of 0 m/s, V0.3 = velocity threshold of 0.3 m/s, V0.5 = velocity threshold of 0.5 m/s, vMaxProRPE = strength estimation under consideration of the perceived exhaustion of the participants, * = significant after Bonferroni correction (threshold = 0.002).

**FIGURE 1:**
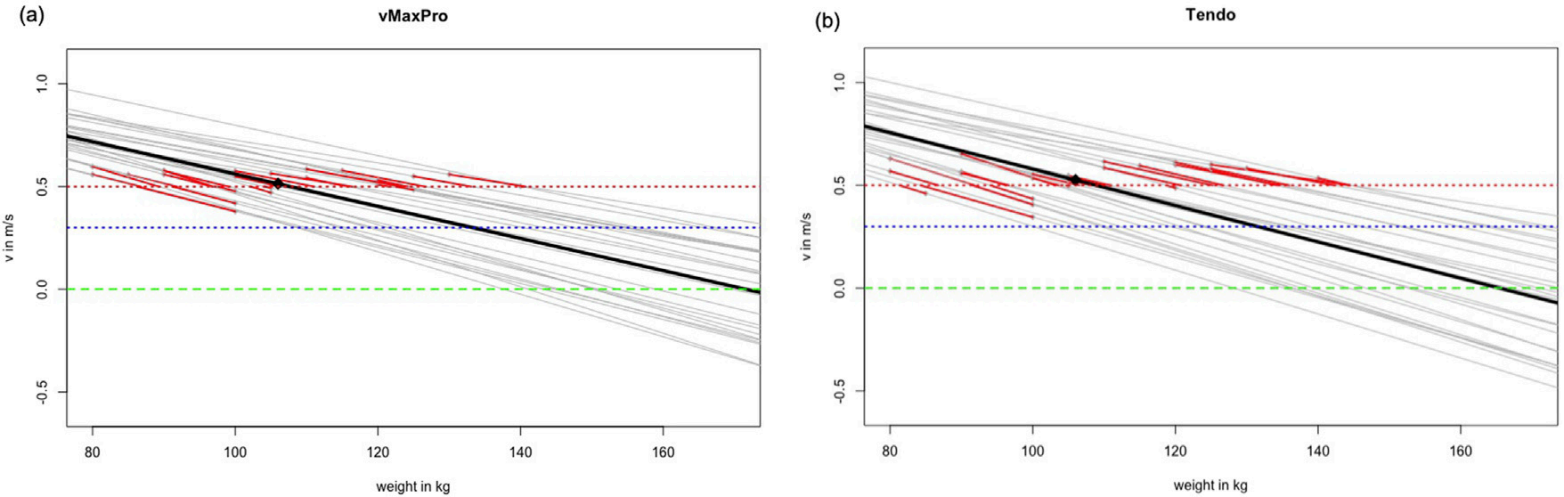
Provides the linear regression calculated from the data points (mean (black) and individual (gray) courses) with different velocity thresholds (green LD0, blue v = 0.3 m/s, and red v = 0.5 m/s dotted lines) to provide the individual errors to the measured 1RM for vMaxPro **(A)** and Tendo **(B)**.

Correlations between the real 1RM and the estimated 1RM independent of the used sensor or model provided high correlation coefficients (r_p_ = 0.82–0.94). With exception of the v = 0.3 m/s model (ICC = 0.36 and 0.40), the relative values determined via ICC and CCC were 0.82–0.93, however, accompanied by a systematic bias for all models (but vMaxProRPE with *p* = 0.08) either under- or overestimating the squatting performance (*p* < 0.001–0.005) with a random error of MAE = 4.32–29.4 kg, MAPE = 4.12–27.5%. Although the v = 0.3 m/s model could be considered an outlier, there was still a random scattering of up to 10% (MAPE = 9.1%), apart from the systematic estimation bias. The results of the agreement analysis are presented in Table 3 and Figure 1.

## 4 Discussion

The study investigated the validity and precision of two commonly used velocity sensors by testing them with elite soccer players. The reliability statistics provided may raise some concerns about their practical usability. With ICCs commonly ranging between 0.65 and 0.80, the provided relative reliability values agree with those of previous research (Dragutinovic et al., 2024; Feuerbacher et al., 2022). Previous biostatistical articles stressed the limited value in assessing measurement errors and recommended quantifying systematic errors as well as random “noise” (Atkinson and Nevill, 1998; Hopkins, 2000). With an MAE of 0.03–0.22 and a corresponding MAPE of up to 7.5% arising from repeating the same procedure, the results question the precision and accuracy as well as repeatability of these devices.

The study design has revealed an unexpected measurement error between the testing devices. Because both velocity devices were attached to the same bar, a minimal testing error was assumed. Agreement ICCs covered a range from small to high, and concordance correlation coefficients between 0.28 and 0.80 were accompanied by MAEs ranging from 0.03 to 0.13, corresponding to a tracking error of up to 11%. Repeating the same testing caused an unacceptable error (from a practical perspective), while both attached sensors led to the same velocity. Referring to Gauss propagation of uncertainty (Srinivasan et al., 2012), suggesting an accumulation of measurement errors from different sources when calculating a 1RM prediction, the internal and external validity of the measured load–velocity profiles must be questioned. Even though some previous studies explored the agreement between different motion-capturing system devices (Feuerbacher et al., 2022), no previous studies quantified the accompanying random error.

### 4.1 Predicting one-repetition maximum using the load–velocity profile

Indentifying the origin of such measurement errors is relevant. If they were measured between two different trials within one device, the refer to the intraday reliability, while if their occurrence between two different devices within one repetition could be viewed as a type of objectivity. The resulting values provide a corridor in which the true velocity of the bar can be considered. Accordingly, the used devices do not allow a clear velocity determination. Based on the present data, it is not possible to state which of the two velocity trackers measured the actual velocity of the bar or if both produced an unacceptable measurement error. Although previous studies focused on ICCs to determine reliability and did not quantify the random or systematic error, the relative reliability results of the present study are in accordance with other studies.

Several factors must be considered when interpreting this testing error. An important aspect is that the prediction model precision depends on the 1RM bar velocity used for the regression (Weakley et al., 2021). While comprehensive from a pure physical position (action = reaction, when exerting maximal force, the bar must not be further moved), many studies used the v = 0 m/s in the regression model to estimate the 1RM load. While Gomes et al. (2024) and Fitas et al. (2024) proposed the LD0 method (v = 0 m/s threshold) to be valid and reliable in the back squat, this assumption caused a huge systematic overestimation of the 1RM in the present study. These possibly explain huge prediction errors in, for example, the Hughes study as well. Thus, because the v0 method seems inappropriate for practical use, Guppy et al. (2023) reported 1RM velocity in the squat with v = 0.32 m/s, which was applied to the present data (v = 0.3 m/s). Although this threshold might apply to other populations, this model systematically overestimated the predicted 1RM by approximately 35 kg, which seems in accordance with Banyard et al. (2017). The v = 0.3 m/s might not be suitable in the present study, which could be attributed to the age of the included participants, as only adolescents (U17 and U19 athletes) were included. In the last step, the v = 0.5 m/s threshold also showed a systematic bias. In contrast, only the vMaxPro internal calculation software with the inclusion of the RPE (Helms et al., 2017) improved the predicted mean value. Even though the mean difference decreased to 2.24 kg (*p* = 0.08), there was still an unsystematic prediction error of 4.42%. In the worst case (MPE), the prediction model still failed with 17% of the real tested maximal strength. These unsystematic errors are mostly neglected in ICC statistics but might meaningfully impact individual athletes. Therefore, individual courses must be considered when investigating the practical applicability.

While all available models (generalized as well as individualized v1RM models) produced unacceptable prediction errors (Fernandes et al., 2021; Jukic et al., 2022), the systematic overestimation as well as relative reliability value concerns are only one part of the problem. Frequently reported systematic bias (systematic over- or underestimation of the predicted 1RM) would be a solvable problem by multiplying the mean with a fixed factor to improve the predictability. The random scattering of individual measures around the mean value can be described as the random bias or “noise” (Hopkins, 2000). Apart from this, authors frequently used Pearson correlation, aiming to validate velocity-based 1RM estimates by correlating it with the measured 1RM. The limited validity of using correlation coefficients to explore agreement between the estimated and measured strength value was reported as early as 1989 by Lin (1989). The present study’s data underline the missing value of frequently used correlation coefficients (Banyard et al., 2017; Kilgallon et al., 2022; Ruf et al., 2018) to assess agreement (Bland and Altmann, 1986; Doğan, 2018; Giavarina, 2015; Lin, 1989). Table 3 provides these with r_p_ = 0.79–0.92, while the CCC drops partially to 0.26. The mean of the percentage random scattering reached 36%. As requested by Guppy et al. (2023), the present study provides a detailed agreement analysis that determined measurement errors between trials and sensors that might or might not contribute to a discrepancy between the measured and predicted 1RM, underlining the authors’ conclusion:

“*When the current body of scientific literature is taken collectively, it seems relatively clear that although using a load-velocity profile to predict 1RM strength on a day-to-day basis presented a theoretically promising programming strategy, in practice, it is not yet feasible and can result in the mis-programming of training loads.*”

Especially in high-level performance athletes, it seems careless to use prediction models with a mean measurement error of approximately 10%, especially because maximal deviations led to more than 25% individual prediction errors. Athletes will be exposed to training loads meaningfully exceeding their physical capacities, causing a substantial injury risk.

### 4.2 Practical relevance

Referring to the current guidelines of sports science (French and Torres Ronda, 2022), scientists should provide athletes and coaches with helpful content to improve performance. Focusing on relative reliability values and mean differences/systematic bias while neglecting individual courses not only provides misinformation but also enhances the injury risk by misinterpreting statistical key parameters, which must be avoided. If 1RM prediction should be applied to load control in sports practice, the random error, thus individual scattering around the systematic bias value, must be minimized to provide benefits. To counteract those limitations, machine learning approaches were proposed by Balsalobre-Fernández and Kipp (2021). However, because the first approaches also focus on correlation analyses and mean differences, advanced statistics and concrete approaches must be systematically investigated to determine which parameters moderate the outcome of the learning system. However, the risk of misinterpreting statistical key parameters still applies to machine learning approaches.

Another limitation of the practical relevance is that current approaches use fixed percentage weights calculated from a previously determined 1RM to extrapolate the 1RM of participants included in the calculation model. Focusing on regression models of included participants does not provide generalizable prediction models for participants not included in this regression model. Due to the outlined limitations, it must be recommended to assess maximum strength using traditional methods (Guppy et al., 2023) because traditional 1RM testing was shown to be reliable (Grgic et al., 2020). Before applying velocity approaches, more research is necessary to develop valuable estimation protocols. However, the present results agree with the most recent literature, concluding the determination of the 1RM squat via the velocity profile is not a viable option for estimating the 1RM in the free-weight back squat (LeMense et al., 2024).

### 4.3 Limitations

Some study limitations must be acknowledged. First, the sample participants, 25 male elite youth soccer players, originate from a highly specific population. Although linear load–velocity relationships were assumed for both men and women (Loturco et al., 2021; Ruf et al., 2018), the influence of sex cannot be excluded, limiting the generalizability of the results. Another variable of interest is the training level. Participants using approximately 100 kg in the back squat might, on the one hand, not considered elite strength athletes; however, as Hughes et al. (55) reported heteroscedasticity for the 1RM estimation, study results might have limited transferability to untrained participants (in which maximal strength testing is contraindicated in general (Warneke et al., 2023)) or to elite powerlifting athletes who would have significantly higher 1RM values. Furthermore, as pointed out by Weakley et al. (2021), selecting the appropriate velocity threshold used in the regression model has a significant impact on the results and prediction error. Even though this study used evidence-based thresholds (v = 0 m/s, v = 0.3 m/s, and v = 0.5 m/s), none of the considered models seemed suitable to provide a testing error that could be accepted for practical applications. Even though the random error—which is of the highest importance for practical usage—would not be reduced, it is possible that the included thresholds were not appropriately chosen to estimate the 1RM. Nevertheless, Weakley et al. (2021) and Fernandes et al. (2021) showed similar prediction errors for individualized and generalized 1RM velocity assumptions.

The included participants were able to lift the weight of 90% 1RM with approximately v = 0.6 m/s, which is above the value stated by Guppy et al. (2023) with v = 0.47 m/s. Therefore, it is possible that the participants did not perform their repetition with the highest possible effort, biasing the calculation model.

Investigators manually extracted and fitted the data from the velocity devices into the MS Excel sheets, which could cause extraction errors. As no automatic extraction option was available, tables were double-checked by another author. Nevertheless, human extraction errors could cause errors in results that remained undetected. These, as well as further random error sources such as unsystematic fatigue throughout the testing, intersubject recovery differences, or other unsystematic sources of errors related to the participants and investigators, seem to be potential explanatory approaches for the random error. Which exact factors cause random errors in load–velocity profiles seems a matter of debate that cannot be explained within this study and calls for future research. Further investigations to assess the systematic and random error require larger sample sizes to reasonably apply multivariate analysis approaches such as analyses of variance (number devices × quantity of testing occasions × quantity of loads).

## 5 Conclusion

In the tested population, neither the intraday reliability nor the device accuracy provided sufficient results to be reasonably integrated into training routines. Using the internal vMaxPro software and linear regression models, the agreement between the previously determined 1RM and the predicted 1RM was low, with systematic and random measurement errors prohibiting the application in training practice to avoid overuse injury. The results call for more careful data handling in future validity and reliability studies to provide relevant training recommendations that account for individual courses, as the exclusive focus on means, standard deviations, and relative reliability values might be of limited interest to coaches, athletes, and clinicians. To investigate the potential of incorporating velocity profiles for 1RM prediction into training practice, further research using advanced analysis methods and/or machine learning approaches is necessary to determine suitable models. While the precision of the load–velocity profile estimated 1RM is somewhat improved by constructing the profile with a final submaximal load that is close to the 1RM (i.e., 90% 1RM), ultimately, the precision of the estimation was still unacceptable in free-weight exercises when compared to direct measurement of 1RM in the presented data.

## Data Availability

The raw data supporting the conclusions of this article will be made available by the authors, without undue reservation.
